# Targeting of *RET* oncogene by naphthalene diimide-mediated gene promoter G-quadruplex stabilization exerts anti-tumor activity in oncogene-addicted human medullary thyroid cancer

**DOI:** 10.18632/oncotarget.10105

**Published:** 2016-06-16

**Authors:** Alessia Lopergolo, Rosalba Perrone, Monica Tortoreto, Filippo Doria, Giovanni L. Beretta, Valentina Zuco, Mauro Freccero, Maria Grazia Borrello, Cinzia Lanzi, Sara N. Richter, Nadia Zaffaroni, Marco Folini

**Affiliations:** ^1^ Department of Experimental Oncology and Molecular Medicine, Fondazione IRCCS Istituto Nazionale dei Tumori, 20133, Milano, Italy; ^2^ Department of Molecular Medicine, University of Padua, 35121, Padova, Italy; ^3^ Department of Chemistry, University of Pavia, 27100, Pavia, Italy

**Keywords:** gene promoter, G-quadruplex, medullary thyroid cancer, naphthalene diimide, RET oncogene

## Abstract

Medullary thyroid cancer (MTC) relies on the aberrant activation of RET proto-oncogene. Though targeted approaches (i.e., tyrosine kinase inhibitors) are available, the absence of complete responses and the onset of resistance mechanisms indicate the need for novel therapeutic interventions. Due to their role in regulation of gene expression, G-quadruplexes (G4) represent attractive targets amenable to be recognized or stabilized by small molecules. Here, we report that exposure of MTC cells to a tri-substituted naphthalene diimide (NDI) resulted in a significant antiproliferative activity paralleled by inhibition of RET expression. Biophysical analysis and gene reporter assays showed that impairment of RET expression was consequent to the NDI-mediated stabilization of the G4 forming within the gene promoter. We also showed for the first time that systemic administration of the NDI in mice xenotransplanted with MTC cells resulted in a remarkable inhibition of tumor growth *in vivo*. Overall, our findings indicate that NDI-dependent RET G4 stabilization represents a suitable approach to control RET transcription and delineate the rationale for the development of G4 stabilizing-based treatments for MTC as well as for other tumors in which RET may have functional and therapeutic implications.

## INTRODUCTION

Medullary thyroid cancer (MTC) is a malignant disease arising from neural crest-derived thyroid parafollicular C cells [1, 2]. It may occur both sporadically (75 % of cases) and in a context of inherited tumor syndromes (25 % of cases), presenting as multiple endocrine neoplasia (MEN) type 2A (80 %), MEN2B (5 %), or familial MTC (15 %) [1, 2].

The *RET* (REarranged during Transfection) proto-oncogene is tightly associated with MTC development [3]. *RET*-activating germline point mutations are the driver events in MEN2 syndromes whereas somatic mutations of *RET* proto-oncogene are present in 30-50 % of sporadic MTC [1, 2]. *RET* gene encodes a single-pass transmembrane tyrosine kinase receptor which is expressed in cells deriving from the branchial arches, the neural crest and the urogenital system [3, 4]. By direct phosphorylation of multiple downstream targets, the mutant RET tyrosine kinase receptor controls the proliferation and survival of MTC cells [3, 4].

Total thyroidectomy represents the only curative option for MTC [5]. Unfortunately, most MTCs are diagnosed when the disease is already metastatic and often exhibits resistance to chemo- and radio-therapy [1–5]. Several preclinical studies have provided compelling evidence on the relevance of RET as promising therapeutic target [5, 6]. Though targeted approaches based on the use of tyrosine kinase inhibitors (TKIs), such as vandetanib or cabozantinib, have represented a breakthrough for the treatment of metastatic disease [5], the absence of complete responses in clinical trials highlights the need for treatment optimization. In addition, the occurrence of primary and/or secondary resistance mechanisms, such as the described point mutation in Valine 804 of RET receptor [7], represents a worrisome event for the treatment of advanced MTC with selected TKIs. Therefore, the development of novel therapeutic interventions for the advanced disease is a mandatory issue.

G-quadruplexes (G4s) are peculiar structures that can form in guanine-rich nucleic acids [8, 9]. They are composed of four guanine residues self-assembled into stable planar arrays, known as G-quartets, in which guanines are interconnected by Hoogsteen hydrogen bonds [8, 9]. The stacking of two or more G-quartets results in the formation of a G4 supramolecular structure, which may be stabilized by monovalent cations (e.g., Na^+^; K^+^) and ligands, including small molecules and proteins [8, 9]. Nucleic acid sequences that may fold into G4 structure exhibit a consensus motif (i.e., G4 forming motif), which consists of at least four runs of guanines containing three or more guanine residues (e.g., [G_≥3_N_x_]_≥4_, where N is any nitrogen base and X a number between 1 and 7) [9]. Genome-wide analyses have revealed an extensive representation of G4 forming motifs throughout the human genome [9]. Other than in telomeres, these motifs are particularly abundant within the replication origins and, at gene level, within the promoter regions flanking the transcription start site (TSS), the 5′ untranslated region (UTR) and the 5′ end of the first intron [10, 11]. Conversely, G4 forming motifs are poorly represented at the 3′-UTRs and almost absent in exons [10, 11]. Furthermore, oncogene rather than tumor suppressor gene promoters are chiefly enriched in these motifs, thus suggesting an evolutionary selection for G4 structures based on gene function [12].

Although the physiological role of G4s still needs to be fully elucidated, a growing body of evidence points them out as attractive targets to defeat cancer [13]. In this context, G4s exhibit a variety of well-defined structural elements such as molecularity, strand and loop orientation (generally defined as topology), loop composition/length and groove features [8–12]. Such a diversity of G4 structural elements provides, at least in principle, a repertoire of specific druggable sites amenable to be efficiently recognized and stabilized by small molecules (i.e., G4-ligands or G4 stabilizing agents) for therapeutic purposes [13, 14].

This evidence has indeed fuelled the search for compounds able to interact with and to target such secondary DNA structures [14]. A variety of G4-stabilizing small molecules identified during the last decade are currently considered as fascinating weapons to therapeutically operate at genomic level and represent an entirely novel, though still challenging, approach to anticancer drug design and development [14]. These agents, belonging to different chemical families [13], have been demonstrated to selectively interact with and efficiently favor the folding or the stabilization of G4 structures in a variety of target nucleic acid sequences [13].

By analogy to other TATA-less promoters (e.g., *MYC, KRAS* and *VEGF* genes), the proximal region upstream the TSS of *RET* gene contains two G-rich boxes that are essential for basal promoter activity and are characterized by a typical G4 forming motif [8, 15, 16]. On the basis of this evidence, we investigated the biological effects of a naphthalene diimide (NDI) derivative, previously identified to act as both G4 ligand [17] and a selective photoreactive warhead targeting G4s [18], in two MTC cell lines. Results showed that the exposure of MTC cells to the NDI derivative resulted in a marked impairment of *in vitro* tumor cell growth, which was tightly associated with a remarkable down-regulation of RET, both at mRNA and protein levels. Such an effect was due to the capability of the compound to stabilize the G4 structure that spontaneously forms within a region proximal to the TSS of the oncogene promoter, as revealed by biophysical and gene reporter assays. Moreover, we also provided evidence that *in vivo* systemic administration of the NDI derivative resulted in growth inhibition of tumors originated from MTC cells xenotransplanted into immunocompromised mice.

Overall, our findings suggest that RET G4 stabilization by small molecules may represent a suitable tool to control oncogene transcription for therapeutic purposes in RET-dependent MTC, and furnish the biological rationale for the development of novel G4 stabilizing-based treatment approaches that could be extended to other oncogene-driven neoplastic diseases.

## RESULTS

### Evaluation of the biological effects of the NDI derivative in MTC cells

**Figure 1:**
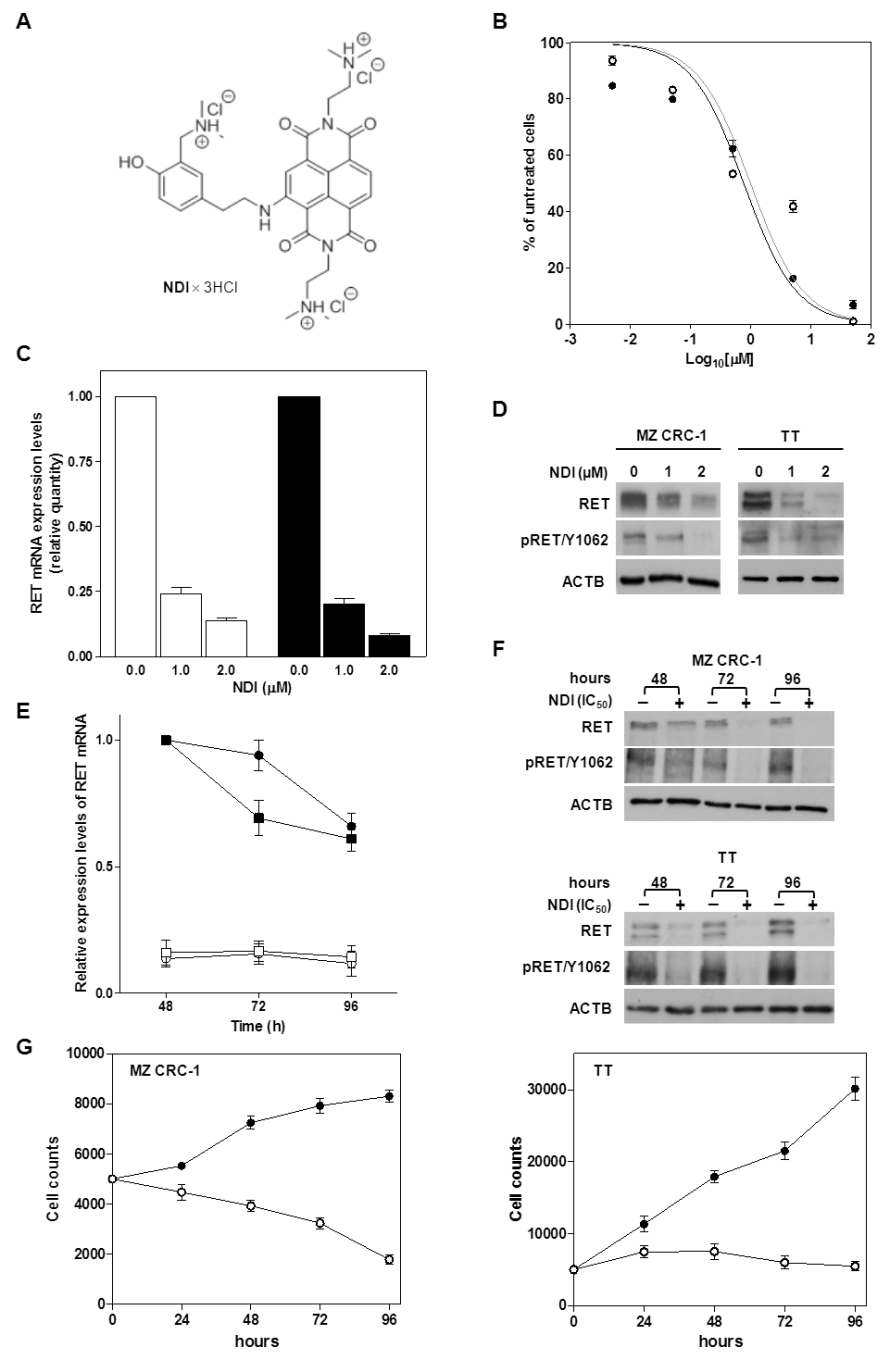
Exposure of MTC cells to the NDI derivative resulted in the impairment of cell growth and in a remarkable down-regulation of RET expression **A.** Chemical structure of the NDI derivative [17]; **B.** Cell growth inhibition curves obtained upon a 48-h exposure of MZ CRC-1 (●) and TT cells (○) to increasing concentrations (Log_10_[μM]) of NDI. Data have been reported as percentage of growing cells in treated *vs.* untreated cells and represent mean values ± s.d.; **C.** Real-time RT-PCR assessment of RET mRNA expression in MZ CRC-1 (white columns) and TT (black columns) cells exposed for 24 h to increasing concentrations of NDI. Quantification of RET mRNA levels was carried out according to the 2^-ΔΔCt^ method. Data have been reported as RET mRNA relative quantity in treated *vs.* untreated cells and represent mean values ± s.d.; **D.** Representative western immunoblotting showing basal and phosphorylated (pRET/Y1062) RET protein amounts in MTC cells exposed for 24 h to the indicated concentrations of NDI. β-actin (ACTB) was used to ensure equal protein loading; **E.** Time-course assessment of RET mRNA expression levels in untreated MZ CRC-1 (●) and TT (■) cells and after the exposure to an equitoxic amount of NDI (MZ CRC-1 (○) and TT (□)). Data have been reported as RET mRNA relative amounts in untreated and NDI-treated cells, according to 2^-Δ*C'*T^ method [39], and represent mean values ± s.d.; **F.** Representative western immunoblotting showing the time-course assessment of basal and phosphorylated (pRET/Y1062) RET protein amounts in untreated and NDI-treated MZ CRC-1 (upper panel) and TT (lower panel) cells; **G.** Time-course evaluation of cell growth in untreated (●) and NDI-treated (○) MZ CRC-1 (left panel) and TT (right panel) cells. Data have been reported as number of growing cells and represent mean values ± s.d.

The biological effects of the NDI derivative (Figure 1A) were investigated in two human MTC cell lines, namely TT cells harboring both a MEN2A-type C634W RET mutation and a tandem duplication of the mutated RET allele [19] and MZ CRC-1 cells harboring the M918T RET mutation found in about 50 % of sporadic MTC and the most common RET mutation in the MEN2B syndrome, associated with an aggressive disease behavior [19].

The capability of the NDI to inhibit the *in vitro* growth of MTC cells was investigated. A 48-h exposure of both MTC cell lines to increasing concentrations of the NDI resulted in a remarkable and concentration-dependent inhibition of cell growth (Figure 1B), with IC_50_ values in the low micromolar range (1.8 ± 0.3 and 1.7 ± 0.2 μM for MZ CRC-1 and TT cells, respectively). The NDI-mediated inhibition of MTC cell growth was associated with a marked down-regulation of RET expression. In particular, real-time RT-PCR analyses showed a nearly complete abrogation of RET mRNA levels in cells exposed for 48 h to 1 μM (0.24 ± 0.03 and 0.20 ± 0.01 relative quantity with respect to untreated cells in MZ CRC-1 and TT cells, respectively) and 2 μM (0.14 ± 0.02 and 0.08 ± 0.006 relative quantity with respect to untreated cells in MZ CRC-1 and TT cells, respectively) of the compound (Figure 1C). The reduction of RET mRNA expression levels was paralleled by the depletion of either the basal and phosphorylated forms of RET protein in both cell lines exposed to the NDI derivative (Figure 1D). Furthermore, the NDI-mediated inhibition of *RET* transcription and reduction of protein amounts was consistently observed up to 96 h in both cell lines treated with an equitoxic concentration (IC_50s_) of the compound (Figure 1E and 1F). Such an effect was paralleled by a pronounced impairment of MTC cell growth over time. In particular, while untreated cells showed an exponential growth (according to their respective doubling times) during the time course of the experiments (Figure 1G), the number of NDI-treated cells was progressively reduced (MZ CRC-1 cells) or remained constant (TT cells) at the different time points (Figure 1G).

To further explore the therapeutic potential of the NDI, we assessed its cytotoxic activity in an *in vitro* model of estrogen-receptor positive breast cancer (BCa), a tumor type for which anti-RET therapy may represent a novel treatment option [20]. Specifically, a 48-h exposure of wild-type RET expressing MCF-7 cells [20] to increasing concentrations of NDI resulted in a dose-dependent inhibition of cell growth (Supplementary Figure S1A), with an IC_50_ value (1.35 ± 0.05 μM) comparable to that obtained in MTC cells. The NDI-mediated impairment of MCF-7 cell growth was paralleled by a concentration-dependent decrease of RET mRNA and protein levels (Supplementary Figure S1B and S1C). In addition, the exposure of MCF-7 cells to a concentration of NDI corresponding to the IC_50_ triggered a persistent inhibition of *RET* transcription over the time course of the experiment (Supplementary Figure S1D), although a noticeable decrease in RET mRNA levels and protein amounts over time was also observed in untreated, exponentially growing cells (Supplementary Figure S1D, S1E and S1F). Nevertheless, a remarkable and time-dependent arrest of cell growth (Supplementary Figure S1F) was appreciable in NDI-treated compared to untreated MCF-7 cells (Supplementary Figure S1F).

To verify whether the NDI-mediated modulation of endogenous RET protein levels was determinant for the antiproliferative activity of the compounds, RET protein/oncoprotein expression was partially restored by transient transfection of plasmids encoding the MEN2A-associated C634R RET (pRc-protoRET^C634R^) variant and the wild-type RET (pRc-protoRET^WT^), respectively, in TT and MCF-7 cells.

Of note, after a 48-h exposure to NDI (IC_50_) a slight, though significant, rescue of cell growth was appreciable in RET- compared to empty vector (mock control)-transfected cells (69 ± 5.5 % *vs.* 54.8 ± 2.0 % and 73.4 ± 10 % *vs.* 54.4 ± 7.5 % for TT and MCF-7 cells, respectively; Supplementary Figure S2A). Furthermore, such a rescue in cell growth was consistently observed up to 96 h in RET^WT^-transfected MCF-7 cells, which showed significantly higher amounts of RET protein, before and after NDI treatment, compared to mock control (Supplementary Figure S2B). This evidence indicates that the ectopic reconstitution of RET may counteract the cell growth inhibitory activity of NDI, thus suggesting that the antiproliferative activity of the compound may be partly limited by the amounts of RET protein.

**Figure 2:**
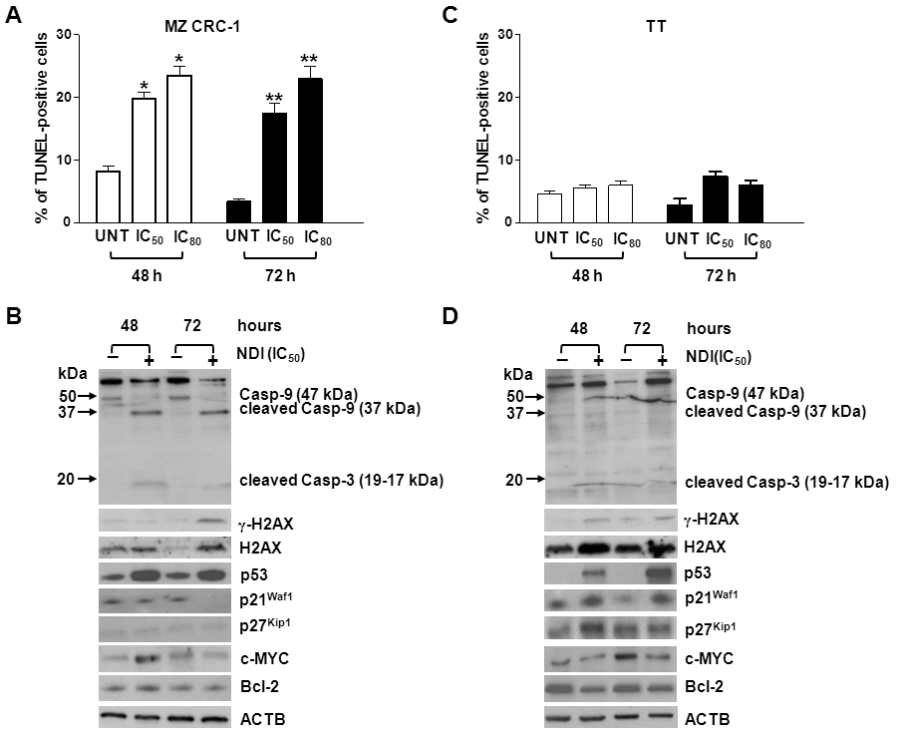
Exposure of MTC cells to NDI resulted in either a cytotoxic or cytostatic effect **A.** and **C.** Quantification of apoptosis induction in MZ CRC-1 (A) and TT (C) cells upon a 48- and 72-h exposure to NDI (IC_50s_ and IC_80s_). Data have been reported as percentage of TUNEL (apoptotic)-positive cells within the overall cell population and represent mean values ± s.d. *P<0.05; **P<0.01; **B.** and **D.** Representative western immunoblottings showing the amounts of apoptosis- and cell cycle-related factors in MZ-CRC1 (B) and TT (D) cells upon a 48- and 72-h exposure to an equitoxic amount (IC_50s_) of NDI. Numbers on the left of each panel indicate the molecular weights (kDa) used as reference for the correct identification of caspase-9 and −3 cleavage products. β-actin (ACTB) was used to ensure equal protein loading.

We have found that NDI exerted a cytotoxic and a cytostatic effect in MZ CRC-1 and TT cells, respectively (Figure 1F). This evidence was further confirmed by the observation that the exposure of MZ CRC-1 cells to the NDI derivative resulted in induction of apoptosis (Figure 2). Specifically, the TUNEL assay revealed a significant increase in the percentage of apoptotic cells upon exposure to the compound for 48 h [19.8 ± 1.0 % (IC_50_) and 25.5 ± 1.5 % (IC_80_); *P*<0.05] and 72 h [17.5 ± 1.5 % (IC_50_) and 23.0 ± 2.0 % (IC_80_); *P*<0.01] compared to untreated cells, in which the apoptotic rate was <10% at both time points considered (Figure 2A). In addition, biochemical analyses revealed a noticeable accumulation of p53 and a remarkable increase in the cleavage of caspase-9 and caspase-3 after a 48- and 72-h exposure to the NDI derivative (Figure 2B). Moreover, a prominent boost of γ-H2AX was appreciable at 72 h in NDI-treated compared to untreated MZ CRC-1 cells (Figure 2B), which is in keeping with the kinetic of apoptotic-induced DNA fragmentation. By contrast, no evidence of apoptosis, in terms of both positivity to TUNEL assay and activation of apoptotic effectors, was observed in TT cells upon exposure the NDI at both time points (Figure 2C and 2D). However, an increase in the levels of γ-H2AX which was associated with a remarkable accumulation of p21^waf1^, a cyclin-dependent kinase inhibitor which mediates p53-dependent cell cycle arrest in response to a variety of stress stimuli, was observed after a 48- and 72-h exposure of TT cells to NDI. In addition, the accumulation of p27^Kip1^, which is known to cooperate with p21^waf1^ in the activation of stress-induced cell senescence [21], was also appreciable in TT cells after a 48-h treatment with NDI (Figure 2D). Similarly, a cytostatic activity associated with a marked accumulation of p21^waf1^ (Supplementary Figure S1E and S1F) was also observed in MCF-7 cells upon a prolonged exposure to NDI. On the contrary, no changes in p21^waf1^ and p27^Kip1^ levels were observed in NDI-treated compared to untreated MZ CRC-1 cells at both time points (Figure 2D). This evidence would indicate that treatment with the NDI triggers an apoptotic cell response in MZ CRC-1 cells, whereas it likely results in a senescent phenotype in apoptosis-deficient TT and MFC-7 cells [22–24].

### The NDI derivative efficiently recognizes and stabilizes the G4 structure within *RET* promoter

**Figure 3:**
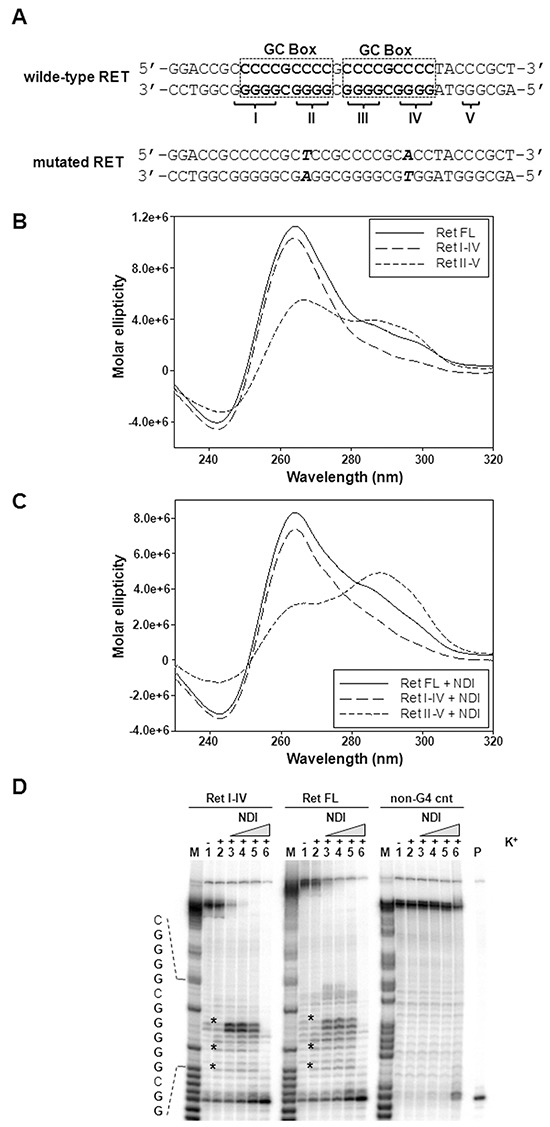
Biophysical analysis of the *RET* proto-oncogene promoter G-rich sequence **A.** Schematic representation of the wild-type G-rich sequence located within the proximal region of *RET* proto-oncogene promoter. Dashed squares delimitate the two GC boxes, according to Guo K. *et al.* [15]. The five G-tracts are labelled by roman numbers. The RET mutated sequence is also shown. Points mutations (G>A and G>T) within G-tract II and IV are highlighted in bold; **B.** Representative CD spectra of the RET FL as well as of RET I-IV and RET II-V truncated oligonucleotides (4 μM) recorded in the presence of 25 mM K^+^. **C.** Representative CD spectra of 4 μM RET oligonucleotides (see panel B) recorded in the presence of 25 mM K^+^ and 16 μM NDI. **D.** Image of a typical *Taq* polymerase stop assay. The RET FL and RET I-IV templates were amplified by the *Taq* polymerase in the absence (lanes 1) and presence of 10 mM K^+^, alone (lane 2) or combined with increasing amounts (0.008, 0.04, 0.2 and 1 μM) of NDI (lanes 3-6). A template (non-G4 cnt) made of a scramble sequence unable to fold into G4 was also used as internal control. Lane M: ladder of markers obtained by the Maxam and Gilbert sequencing carried out on the amplified strand complementary to the template strand. Lane P: unreacted labelled primer. The base sequence of the RET FL template corresponding to the stop sites is shown on the left of the gel image. G bases are shown in bold. Asterisks indicate G4-specific *Taq* polymerase stop sites.

On the basis of the evidence that *RET* promoter contains a typical G4 forming motif (Figure 3A) and on the observed NDI-mediated decrease of RET levels in cancer cells (Figure 1 and Supplementary Figure S1), we investigated by biophysical assays whether the NDI derivative was able to favor the formation and/or the stabilization of G4 structures within the *RET* promoter. Three different RET model oligonucleotides were assayed by circular dichroism (CD): the full-length (FL) sequence comprising five G-tracts (G-tracts I-V), and two truncated sequences corresponding to G-tracts I-IV and II-V, which contain the minimum number of G-tracts required to form a G4 (Figure 3A and Supplementary Table S1). G-tracts I-IV have been reported to form the major G4 structure within the FL RET sequence [15]. In the presence of physiological concentration of K^+^, CD spectra of the tested RET sequences were all distinctive of G4 topologies (Figure 3B). In particular, RET FL and RET I-IV sequences formed a parallel-type G4 structure showing a major positive peak at 263 nm and a negative peak at 242 nm, in accordance with previously reported data showing that G-tracts I-IV formed a parallel G4 structure [15, 16]. RET II-V formed a hybrid-type G4 structure with two positive peaks at around 260 nm and 290 nm and a negative peak at around 240 nm. The stability of these G4s was dependent on the presence of K^+^ (Table 1).

**Table 1:** Melting temperature (T_m_) values of G4-folding oligonucleotides (4 μM), corresponding to G-rich regions of the *RET* promoter, in the presence of increasing concentrations of K^+^ and in the absence (−) or presence (+) of NDI (16 μM)

Sequence	T_m_ values (°C)					
	K^+^ 10 mM		K^+^ 25 mM		K^+^ 100 mM	
	−	+	−	+	−	+
**RET FL**	57.7 ± 0.1	>95	82.0 ± 2.2	>95	>95	>95
**RET I-IV**	58.2 ± 0.1	>95	64.7 ± 0.2	>95	81.9 ± 2.0	>95
**RET II-V**	64.9 ± 1.3	>95	67.2 ± 0.6	>95	77.6 ± 1.8	>95

Data are reported as mean values ± standard error. P values <0.0001.

Upon addition of the NDI, CD analysis showed no major conformational variation for RET FL and RET I-IV, while RET II-V inverted the intensity of peaks at 265 and 290 nm (Figure 3C); however, all RET G4s displayed a remarkable increase in their stability (T_m_>95 °C, Table 1 and Figure 3B and 3C) with significant variation of the T_m_ recorded in the presence *vs.* the absence of the compound at all tested K^+^ concentrations (Table 1). To allow assessment of the best stabilized sequence, NDI/DNA ratio was next set to 1 to avoid reaching T_m_ values above 95°C, which would hinder comparison. In these conditions, RET II-V was stabilized by the NDI to a higher extent compared to RET FL and RET I-IV (5.6°C vs 1.2-1.3°C, Supplementary Table S2). Such a stabilization was partly selective for RET, since the G4s formed by c-Kit1 and c-Myc oncogene promoters were stabilized to a lesser extent than RET II-V (1.5 and 3.6 °C; Table S2).

To confirm the ability of the NDI derivative to stabilize the RET G4 conformation in extended DNA sequences, a *Taq* polymerase stop assay was performed using RET FL and RET I-IV templates (Supplementary Table S1). Specifically, in the presence of K^+^, minor stop sites corresponding to bases right before Gs at the most 3′-end of the G-tracts were visible in both RET templates, but not in a control unable to fold into G4 (see * in Figure 3D, lanes 2). Upon addition of increasing amounts (0.008-1 μM) of the NDI, the intensity of the stop bands greatly increased (Figure 3D, lanes 3-6), indicating an effective stabilization of the G4 structures by the compound even in an extended DNA template. Remarkably, a high stabilization of the structure was achieved at nM concentrations of the compound (Figure 3D, lanes 3-5), whereas only at 1 μM, the highest NDI tested concentration (Figure 3D, lanes 6), *Taq* polymerase activity was inhibited non-specifically, as revealed by the occurrence of a faint stop site also on the control template (Figure 3D, lanes 6).

**Figure 4:**
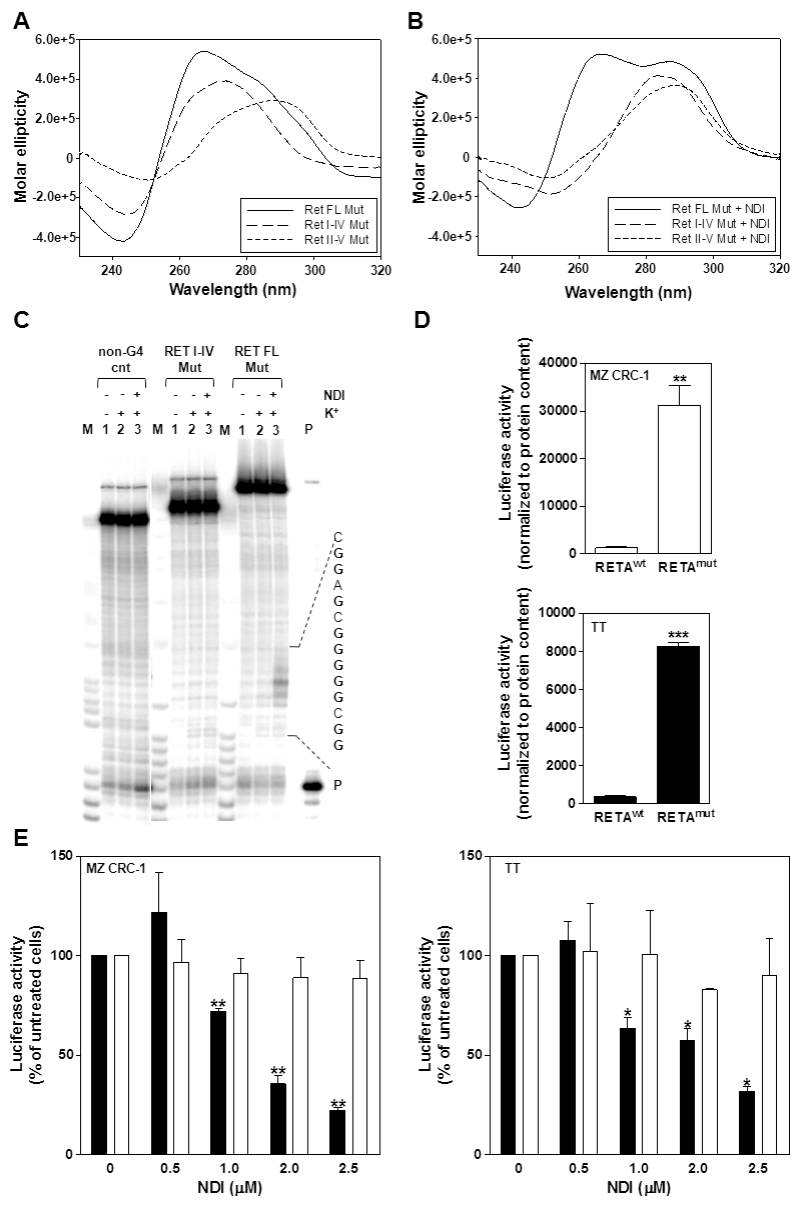
Biophysical analysis of the mutated *RET* promoter sequence **A.** Representative CD spectra of the RET FL Mut, RET I-IV Mut and RET II-V Mut oligonucleotides (4 μM) recorded in the presence of 100 mM K^+^; **B.** Representative CD spectra of 4 μM RET oligonucleotides recorded in the presence of 100 mM K^+^ and 16 μM NDI; **C.** Image of a typical *Taq* polymerase stop assay. The RET FL Mut and RET I-IV Mut templates were amplified by the *Taq* polymerase in the absence (lanes 1) and in the presence of 10 mM K^+^, alone (lanes 2) or combined with 0.2 μM NDI (lanes 3). Lane M: Ladder of markers; P: unreacted labelled primer. The base sequence of the RET FL template corresponding to the stop sites is shown on the right of the gel image. G bases are shown in bold; **D.** Quantification of basal luciferase activity in MZ CRC-1 and TT cells following transfection with pGL3-RETA^wt^ or pGL3-RETA^mut^ vectors. Data have been reported as luciferase activity upon normalization to protein content and represent mean values ± s.d. **P<.01; ***P<.001; **E.** Assessment of luciferase activity in pGL3-RETA^wt^ (black columns)- and pGL3-RETA^mut^ (white columns)-transfected MTC cells exposed to the indicated amounts of NDI. Data have been reported as relative luciferase activity in treated *vs.* untreated cells and represent mean values ± s.d. *P<.05; **P<.01.

Next, two point mutations (G>A and G>T), the position of which was selected on the basis of the evidence that G residues 3, 8, 13 and 18 within guanine runs II and IV are involved in the formation of G-tetrads [15], were inserted (Figure 3A) and their effect on G4 formation was tested in three templates (RET FL Mut, RET I-IV Mut and RET II-V Mut, Supplementary Table S1). Spectroscopic analyses showed that, even in the presence of K^+^ 100 mM, the RET I-IV Mut and RET II-V Mut oligonucleotides essentially failed to fold into a canonical G4 conformation and that the RET FL Mut sequence was highly impaired in its folding because, while still showing a G4-like CD signature, the molar ellipticity was dramatically decreased (Figure 4A). In the presence of the NDI, the shoulder at 290 nm slightly increased in the RET FL Mut sequence, whereas no G4 signatures were yet appreciable in the RET I-IV Mut and RET II-V Mut oligonucleotides (Figure 4B). However, the RET FL Mut sequence was only mildly stabilized by the compound (T_m_ 67°C), especially in light of the significant stabilization imparted to the wild-type sequence (T_m_ > 95 °C) (Supplementary Figure S3). These data were further confirmed by a *Taq* polymerase stop assay. No major products of *Taq* polymerase arrest were observed in all tested sequences in the presence of the NDI, except for a minor stop site appreciable within the RET FL Mut template (Figure 4C). Nonetheless, such a stop site did not prevent the *Taq* polymerase to fully synthesize the whole sequence (Figure 4C, RET FL MUT, lane 3), conversely to what observed with the wild-type RET FL template under the same experimental conditions (Figure 3D). These data indicate that point mutations inhibited to a great extent, though not completely, the capability of the full-length G-rich region of the *RET* promoter to fold in a G4 structure.

### *RET* promoter-driven luciferase activity is markedly inhibited by the NDI derivative

The evidence that the G4-forming region within the *RET* promoter is essential for basal promoter activity [15, 16] and that it can be effectively stabilized by the NDI (Figure 3), led us to hypothesize that the compound could turn-off the expression levels of *RET* in intact cells through a G4 stabilization-based mechanism. To verify such a hypothesis, a gene reporter assay was carried out in MTC cells transfected with a luciferase reporter plasmid bearing the wild-type form of the *RET* promoter (pGL3-RETA^wt^) or its mutated variant (pGL3-RETA^mut^) bearing G>A and G>T point mutations (Figure 3A). Such mutations have been shown to remarkably impair the G4 folding capability of the sequence (Figure 4A–4C), without affecting the consensus sequences for the binding of Sp1/ Sp3 transcription factors (verified by Jaspar 5.0-alpha @ jaspar.genereg.net), which are necessary for *RET* basal promoter activity [25]. Results indicated that transfection of MTC cells with pGL3-RETA^mut^ resulted in a ~20-time increase in luciferase activity compared to cells transfected with the wild-type vector (Figure 4D). This finding is in keeping with CD analysis and shows unambiguously that, under “physiological conditions”, the wild-type G4 forming motif within *RET* promoter spontaneously folds into a G4 structure, which in turn may negatively control the oncogene expression. Moreover, the exposure of cells transfected with pGL3-RETA^wt^ vector to increasing concentrations of NDI resulted in a significant and dose-dependent inhibition of luciferase activity (Figure 4E), which ranged from 71.8 ± 1.5 % to 22.3 ± 1.5 % in MZ CRC-1 cells and from 63.6 ± 5.4 % to 31.8 ± 2.6 % in TT cells, with respect to pGL3-RETA^wt^- transfected cells in the absence of NDI. Conversely, a negligible inhibition of luciferase activity (<12 %) was observed in pGL3-RETA^mut^ vector-transfected MTC cells at all tested NDI concentrations with respect to untreated cells (Figure 4E).

Overall, these findings confirm our initial hypothesis and indicate that NDI-mediated down-regulation of RET in MTC cell lines occurs as a consequence of the G4 stabilizing capabilities of the compound.

### The NDI derivative affects the *in vivo* tumorigenic potential of MTC cells

In the final step of the study, the *in vivo* antitumor activity of the NDI derivative was evaluated in RET-dependent MTC models obtained following xenotransplantation of MZ CRC-1 and TT cells into immunocompromised mice [26]. Dosing regimen and treatment schedule were chosen on the basis of preliminary dose optimization studies carried out by taking into account of available information present in the literature for a compound belonging to the same chemical family [27].

**Figure 5:**
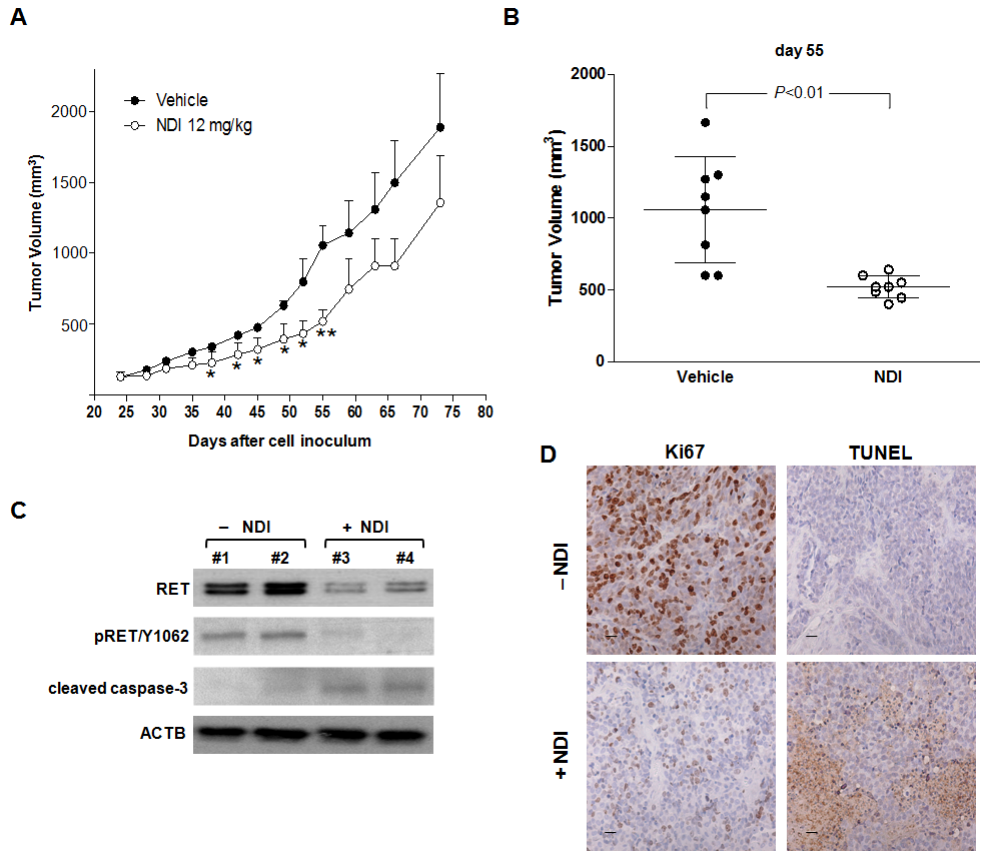
The NDI derivative inhibits MTC tumor growth *in vivo* **A.** Growth curves of MZ CRC-1 tumors in vehicle-treated mice (●) and upon i.p. administration of 12 mg/kg NDI derivative (○). Data have been reported as average tumor volume (mm^3^) ± S.E.M.; *P<.05, **P<.01; **B.** Tumor volume distribution in vehicle- and NDI-treated animals at day 55, at which the maximum TVI% was observed; **C.** Representative western immunoblotting showing basal and phosphorylated (pRET/Y1062) RET as well as cleaved caspase-3 protein amounts in MZ CRC-1 representative tumors grown *in vivo* and collected at day 55; **D.** Representative images showing the immunohistochemical staining for Ki67 (cell proliferation marker) and TUNEL (apoptosis marker) in MZ CRC-1 tumor tissues from control and treated mice, collected at day 55. Scale bar: 30 μm; magnification ×40.

Upon systemic administration, the compound was well tolerated without any appreciable sign of general toxicity and with a restrained effect (<15%) in terms of average body weight loss. Interestingly, a significant tumor growth delay was observed in animals that had received 12 mg/kg i.p. of NDI, compared to vehicle-treated mice (Figure 5A and Supplementary Figure S4A), with a maximum tumor volume inhibition (TVI) of 50% at day 55 (mean TV 1,057 ± 166.6 and 520.4 ± 35 mm^3^ in control and treated groups, respectively; *P*<.01) and of 37% at day 58 (mean TV 662 ± 87 and 416 ± 52 mm^3^ in control and treated groups, respectively; *P*<.05) in MZ CRC-1 and TT xenografts, respectively (Figure 5B and Supplementary Figure S4B). Moreover, the biochemical assessment of the *in vivo* drug-mediated effects showed a marked reduction of the basal and phosphorylated forms of RET protein and an increase in cleavage of caspase-3 in tumors explanted from NDI-treated mice with respect to controls (Figure 5C), thus providing evidence of pharmacodynamic activity and further confirming the *in vitro* observations. Furthermore, a remarkable reduction in the immunohistochemical staining for the proliferation marker Ki67 paralleled by a pronounced increase in the positivity to TUNEL coloration was appreciable in MZ CRC-1 tissue sections from treated animals compared to controls (Figure 5D).

## DISCUSSION

*RET* oncogene-expressing MTC has been reported to retain dependence on RET activity for the maintenance of the malignant phenotype (i.e., oncogene addiction [28]) and, consequently, RET receptor represents an ideal therapeutic target. Its selective abrogation has been indeed actively pursued and several types of molecular weapons have been developed to affect RET expression or activity for therapeutic purposes in thyroid cancer [4]. In this context, the development of TKIs has represented a major progress for the treatment of the metastatic disease [5], though the lack of complete clinical responses and the onset of drug resistance highlight the dire need of ameliorating the therapeutic interventions for the advanced disease [4, 5].

In the present study, we showed that the exposure of *RET* oncogene mutant-expressing MTC cell lines to an NDI-based G4 ligand resulted in pronounced down-regulation of RET expression at mRNA and protein level, as a consequence of the capability of the NDI derivative to stabilize the G4 structure that spontaneously forms within *RET* promoter [15, 16]. Our results support previous observation showing that the RET G4 structure is actually amenable of ligand-mediated stabilization. Specifically, it has been reported that in the presence of monovalent cations the G4-stabilizing agents telomestatin –a macrocyclic natural product isolated from *Streptomyces anulatus* and formerly tested for its capability to stabilize G4 structures at telomeric level - was able to efficiently stabilize the G4 structure in a synthetic oligonucleotide carrying the *RET* promoter sequence [15]. In addition, it has been recently reported that NSC194598 (an indeno[1,2,3-de]quinazoline derivative) and berberine (a natural alkaloid containing a planar isoquinoline aromatic ring) interfered with the oncogene expression and impaired the growth of TT cells cultured *in vitro* by favouring the stabilization of RET G4 and likely preventing the correct assembly of the transcription complex at gene promoter level [29, 30].

In our experimental conditions, the NDI-mediated decrease in RET levels was tightly associated with a marked impairment of MTC cell growth, which in turn was rescued, though partially, by the ectopic expression of RET protein. Such a partial rescue in the phenotype may reside in the multiple targeting properties of the NDI derivative. In fact, the ligand showed to markedly inhibit the growth of melanoma cells through its capability to efficiently interact with telomeric G4s [17] and it displayed fairly good binding properties for G4 structures forming within the promoter regions of *MYC* and *KIT* (Supplementary Table S2, [31]) as well as of *BCL2* [31], other than *RET*. Nonetheless, its capability to inhibit the expression levels of each oncogene in living cells was not observed straightforwardly, being rather dependent on the cell context [31]. In fact, data reported in the present study show that the same molecule markedly impaired RET expression levels in MTC cell lines with a negligible or even contrasting effect on BCL2 and MYC protein amounts (Figure 2B). However, we cannot currently rule out that the phenotype observed in NDI-treated MTC cells may be the result of ligand-mediated multiple action on additional G4 targets (e.g., KIT and telomeres [17, 31]), other than RET, that may be relevant for the disease [32, 33]. In this regard, it should be taken into account that *i)* cell factors (e.g., specific G4 interacting proteins) or cell conditions (e.g., chromatin status or transcriptional activity) may impinge on the ligand/G4 interaction, even in a cell-type dependent manner; *ii)* G4 structures with similar topologies can be amenable of stabilization by the same ligand and that *iii)* the vast majority of ligands reported thus far to interact with promoter G4s were primarily conceived as telomeric G4 ligands (e.g., telomestatin and RHPS4) [13, 15]. Nonetheless, pieces of evidence indicate that the “promiscuous” nature (i.e., the capability to interacts with multiple G4 targets) of some G4 ligands [11, 13, 31, 34, 35] may be of therapeutic value, as it has been recently shown for an NDI derivative able to simultaneously target the G4 within *BCL2* and *KRAS* gene promoters in an *in vivo* model of pancreatic cancer [35].

A remarkable apoptotic response was observed in NDI-treated MZ CRC-1 cells, whereas no apoptosis induction was detected in TT cells following the exposure to an equitoxic amount of the compound. By contrast, NDI-treated TT cells were characterized by a durable cell growth arrest, which was associated with increased levels of markers usually connected to cell senescence, such as the accumulation over time of the cyclin-dependent kinase inhibitor p21^waf1^ and of γ-H2AX. Such a different response of MTC cells to NDI treatment may be accounted by the evidence that TT cells are basically more refractory than MZ CRC-1 cells to different types of apoptotic stimuli [22, 23]. In keeping with this is our observation that MCF-7 cells, which are resistant to apoptosis induction due to the lack of caspase-3 [24], behaved similarly to TT cells upon the exposure to NDI. By analogy, we previously showed that the same derivative contributed to a senescent phenotype by inducing a prolonged cell growth arrest and a time-dependent increase in the expression levels of p21^waf1^ in an *in vitro* model of human melanoma cells [17].

Finally, and most importantly, we here reported that, upon systemic administration, the NDI derivative retained its capability to interfere with RET expression, to remarkably inhibit tumor growth and to induce apoptosis in MTC tumors grown *in vivo* without apparent sign of general toxicity. This evidence contributes to underscore the therapeutic potential of *in vivo* G4 stabilization-based approaches as well as to furnish indication on the safety profile of G4 interacting molecules.

Overall, our data support the notion that small molecule-mediated *RET* G4 stabilization may represent a valid molecular approach for the therapeutic regulation of RET signaling in MTC and contribute to furnish the biological rationale for future development of novel G4 stabilizing-based treatment options for MTC or other neoplastic diseases, such as breast cancers [36] and neuroblastoma [37] for which RET may represent a suitable therapeutic target.

## MATERIALS AND METHODS

### Cell lines and reagents

The human TT cell line, derived from the primary tumor of a sporadic MTC, was purchased from the American Type Culture Collection (LGC Standards S.r.l., Sesto S. Giovanni, Italy). The MZ CRC-1 cell line, derived from the malignant pleural effusion of a patient with a metastatic MTC, was kindly provided by Dr. Robert Hofstra, University of Groningen, The Nederlands [22]. The MCF-7 breast cancer cells were purchased from LGC Standards S.r.l. The cell lines were tested for the absence of *Mycoplasma* fortnightly, monitored periodically for DNA profile of short tandem repeats by the AmpFISTR Identifiler PCR amplification kit (PN4322288, Applied Biosystems/Life Technologies Italia, Monza, Italy). MTC cells were also periodically tested for expression/constitutive activation of RET by western blotting. Cells were maintained in the logarithmic growth phase in 5% CO_2_ at 37°C using appropriate culture media supplemented with 10% fetal bovine serum.

The NDI derivative was prepared according to our previously reported synthetic protocol [17] and purified by preparative HPLC, using a C-18 reverse phase column (OBD-Sun Fire Prep C-18 5μm, 100 × 30 mm, waters eluent CH_3_CN: H_2_O 0.1% TFA), with ≥ 99.9% purity (evaluated by two different HPLC protocols, using a XSelect HSS C18 2.5 μm, 50 × 4.6 mm, waters column; see Supplementary Figure S5 and S6 for details). Addition of 1 ml HCl 1 M solution to 20 ml of the resulting eluate from preparative HPLC, followed by solvent evaporation under vacuum and lyophilisation, afforded the final NDI as hydrochloride (NDIx3HCl, Figure 1A) red powder. The compound was dissolved in DMSO (Sigma-Aldrich S.r.l., Milano, Italy) and stored as stock solution at −20°C. Working solutions of the NDI at the appropriate concentration were prepared in cell culture media just before use.

Mouse monoclonal antibodies anti-p53 (Abcam, Cambridge, UK), anti-p21^waf1^ (NeoMarkers, Fremont, CA, USA) and anti-Ki67 (MIB-1, Dako Italia S.p.A, Cernusco S/N, Italy) along with rabbit polyclonal antibodies anti-Ret (H-300), anti-phospho-Ret/Y1062 (Santa Cruz Biotechnology, Santa Cruz, CA, USA), anti-caspase-9, anti-cleaved caspase-3/Asp175 and anti-c-Myc (Cell Signaling Technology, Danvers, MA, USA), anti-p27^Kip1^, anti-Bcl-2, anti-H2AX, anti-γ-H2AX and anti-β-actin (Abcam.) were used in the study.

Oligonucleotides used for the biophysical analysis are reported in Table S1 and were purchased from Sigma-Aldrich S.r.l. T4 polynucleotide kinase was from Fermentas (Milan, Italy), [γ-^32^P]ATP was from Perkin Elmer (MA, USA).

### *In vitro* studies

For the assessment of NDI-mediated effect on cell growth, cells were seeded at the appropriate density in 6-well plates and exposed to increasing concentrations (0.005, 0.05, 0.5, 5 and 50 μM) of freshly dissolved compound. After a 48-h exposure to the NDI, cells were collected and counted in a particle counter (Coulter Counter, Coulter Electronics, Luton, UK). The IC_50_ and IC_80_ (drug concentration inhibiting cell growth by 50 % and 80 %, respectively) values for each cell line were calculated from dose-response curves. In the medium-term cell growth assay, cells were seeded at the appropriate density in 6-well plates, exposed to a concentration of NDI corresponding to the IC_50_ for 24, 48, 72 and 96 h. Adherent cells were then collected and counted as described above.

Apoptosis was assessed by the terminal deoxynucleotidyl transferase dUTP nick-end labelling (TUNEL) assay using the *In situ* Cell Death Detection Kit, POD (Roche Diagnostic, Monza, Italy). Briefly, NDI-treated cells were harvested at the indicated time points and fixed in 4% paraformaldehyde for 45 min at room temperature. After rinsing with phosphate buffered saline (PBS), the cells were permeabilised in a solution of 0.1 % Triton X-100 in 0.1 % sodium citrate for 2 min in ice. Samples washed with PBS were then incubated in the TUNEL reaction mixture (Boehringer Mannheim, Mannheim, Germany) for 1 h at 37 °C in the dark, and after rinsing with PBS they were suspended in PBS and analyzed by a FACScan cytofluorimeter (BD Biosciences, Milano, Italy). The results were expressed as the percentage of TUNEL-positive cells in the overall cell population.

Details on the procedure used for the ectopic over-expression of wild-type or mutant RET [38] may be found in the Supplementary Information.

### Real-time RT-PCR

Total RNA was isolated from untreated and NDI-treated cells using Qiagen RNeasy Mini Kit (Qiagen S.r.l., Milano, Italy) and DNase I-digested. The expression levels of *RET* mRNA were assessed by real-time RT-PCR by TaqMan^®^ Assay (Hs01120030_m1; Applied Biosystems/Life Technologies Italia). Amplifications were run on the 7900HT Fast Real-Time PCR System (Applied Biosystems/Life Technologies Italia). Data were analyzed by SDS 2.2.2 software (Applied Biosystems/Life Technologies Italia) and reported as relative quantity with respect to a calibrator sample (untreated cells) using the 2^-ΔΔCt^ method [39], where Ct represents the threshold cycle. RNase P (PN4316844) was used as normaliser.

Data obtained in the time-course assessment of RET mRNA levels have been reported according to the 2^-ΔC'T^ method, where ΔC'_T_ = C_T, time x_ - C_T, time 0_.(i.e., 48 h after drug exposure) [39].

### Western immunoblotting

Protein levels were assessed by western blot analyses of whole cell lysates prepared according to standard procedures. Proteins (40 μg) were fractioned by SDS-PAGE and transferred onto Hybond nitrocellulose filters (GE Healthcare Italia, Milano, Italy). Filters were blocked in PBS-Tween 20/5 % skim milk and incubated overnight with primary antibodies (see above). The filters were then probed with secondary peroxidase-linked whole antibodies (GE Healthcare Italia) and bound antibodies visualized by SuperSignal® West PICO chemiluminescent detection system (Fisher Scientific, Pittsburgh, PA, USA). Filters were then subjected to autoradiography. An anti-β-actin (ACTB) antibody was used on each blot to confirm equal protein loading.

### Spectroscopic analyses

Oligonucleotides (Supplementary Table S1) were diluted from stock to a final concentration of 4 μM in lithium cacodylate buffer (10 mM, pH 7.4) and, where appropriate, KCl at different concentration (10-25-100 mM). All samples were annealed by heating at 95 °C for 5 min, gradually cooled to room temperature and measured after 24 h. The compound was added after DNA annealing at the final concentration of 16 μM or 4 μM. CD spectra were recorded on a Chirascan-Plus (Applied Photophysics, Leatherhead, UK) equipped with a Peltier temperature controller using a quartz cell of 5-mm optical path length and scanning a speed of 50 nm/min with a response time of 4 sec over a wavelength range of 230-320 nm. The reported spectrum of each sample represents the average of 2 scans at 20 °C and it is baseline corrected for signal contributions due to the buffer. Observed ellipticities were converted to mean residue ellipticity (θ) = deg×cm^2^×dmol^−1^ (mol. ellip.). For the determination of T_m_, spectra were recorded over a temperature range of 20-95 °C, with temperature increase of 5 °C/min. T_m_ values were calculated according to the van't Hoff equation, applied for a two state transition from a folded to unfolded state, assuming that the heat capacity of the folded and unfolded states are equal [40].

### Taq polymerase stop assay

RET Taq primer (Table S1) was 5′-end labeled with [γ-^32^P]ATP using T4 polynucleotide kinase at 37 °C for 30 min. The labeled primer (final concentration 72 nM) was annealed to the template (final concentration 36 nM) in lithium cacodylate buffer (10 mM, pH 7.4) in the presence or absence of KCl 10 mM. Primer extension was conducted with 2U of AmpliTaq Gold DNA polymerase (2U/reaction, Applied Biosystem/Life Technologies Italia) at 60 °C for 30 min. Where specified, samples were incubated with increasing concentration of NDI derivative (0.008, 0.04, 0.2 and 1μM) in the presence of 10 mM KCl, and primer extension was performed as described above. Reactions were stopped by ethanol precipitation and primer extension products were separated on a 15 % denaturing gel, and visualized by phosphorimaging (Typhoon FLA 9000, GE Healthcare Italia).

### Luciferase assay

The reporter plasmid pGL3-RETA^wt^ [41] containing a 162-bp fragment spanning from −108 to +53 relative to the TSS of *RET* promoter, placed upstream of the *Firefly* Luciferase reporter gene, was kindly provided by Dr I. Ceccherini (Istituto Giannina Gaslini, Genua, Italy). The mutant derivative of pGL3-RETA^wt^ vector carrying a mutated sequence of *RET* promoter was generated by polymerase chain reaction (PCR)-based site directed mutagenesis, according to standard procedure. The RET mutated sequences were obtained by inserting two point mutations (G>A and G>T) within the forward primer sequence. Briefly, the PCR reaction was carried out using pGL3-RETA^wt^ vector as template and forward and reverse primers (Supplementary Table S1) bearing, respectively, unique Kpn I and Hind III restriction sites at the 5′ end. The PCR-amplified fragment and pGL3-RETA^wt^ vector were then digested with Kpn I and Hind III restriction enzymes (New England BioLabs Inc., Ipswich, MA) and purified by agarose gel electrophoresis. The gel-purified RETA^mut^ fragment was then inserted into the KpnI/HindIII digested vector to generate the pGL3-RETA^mut^ plasmid. For the assessment of luciferase activity MZ CRC-1 and TT cells were seeded at the appropriate density (0.6×10^5^ cells/well) in culture plates. Seventy-two h after seeding, cells were transfected with 0.5 μg/well of pGL3-RETA^wt^ or of pGL3-RETA^mut^ using Lipofectamine3000™ (Life Technologies) in standard growth medium containing 10 % FBS, according to the manufacturer's protocol. After 48 h, cells were treated for 24 h with increasing concentrations (0.5-2.5 μM) of NDI. Luciferase activity was measured in cell lysates using the Luciferase Assay System (Promega Italia, Milano, Italy), according the manufacturer's instructions, and normalized to total protein content.

### *In vivo* studies

The *in vivo* antitumor activity of the NDI derivative was assessed using 8-10-week old female SCID mice (Charles River, Calco, Italy) xenotransplanted with MZ CRC-1 or TT cells. Experiments were approved by the Ethics Committee for Animal Experimentation of the Fondazione IRCCS Istituto Nazionale Tumori of Milan according to institutional guidelines, in compliance with national and international laws and policies. Mice were maintained in laminar flow rooms under constant temperature and humidity and with free access to food and water. Briefly, exponentially growing MZ CRC-1 (2×10^7^) and TT (3×10^7^) cells were subcutaneously injected into the right flank of mice. Before starting the treatments, animals were randomly assigned to control or treated groups (n=8/group). The NDI derivative was dissolved in PBS at the proper final concentration just before treatment and administered intraperitoneally (i.p.) at 12 mg/kg every two days for twelve times (q2d×12). The compound was administered to animals when tumors reached a volume of approximately 100 mm^3^ after cell injection. Tumor growth in control and treated groups was followed by biweekly measurements of tumor diameters with a Vernier caliper. Tumor volume (TV) was calculated according to the formula: TV (mm^3^) = d2×D/2 where d and D are the shortest and the longest diameter, respectively. Treatment efficacy was assessed as tumor volume inhibition percentage (TVI %) in treated *vs.* control mice, calculated as 100-(mean TV in treated animals/mean TV in controls × 100). The toxicity was evaluated as body weight loss (BWL).

Vehicle- and NDI-treated tumors were explanted at the end of treatments for subsequent analyses. Immunohistochemistry (IHC) was carried out at the institutional IHC facility on formalin-fixed, paraffin embedded (FFPE) tissue sections (4 μm thick). Briefly, immunohistochemical staining for the proliferation marker was performed using an anti-Ki67 antibody, whereas apoptosis was assessed by the *In situ* Cell Death Detection Kit, POD (Roche Diagnostic), according to the manufacturer's protocol. Immunoreactions were developed using the streptavidin-biotin-peroxidase technique, followed by counterstaining with haematoxylin (Ki67 staining) or by anti-fluorescein antibody Fab fragments (TUNEL assay) conjugated with horse-radish peroxidase (POD). Images were acquired (magnification ×40) using a Nikon Eclipse E600 microscope (Nikon, Japan) by the ACT-1 software (Nikon) and processed by Adobe Photoshop Image Reader 7.0.

### Statistical analysis

Data are reported as mean values ± s.d. (if not otherwise indicated) from at least three independent experiments. Two-sided Student's *t* test was used for the statistical evaluation of data. *P* values <0.05 were considered statistically significant.

## SUPPLEMENTARY MATERIALS FIGURES AND TABLES


