# Supplementary material for: Targeting of RET oncogene by naphthalene diimide-mediated gene promoter G-quadruplex stabilization exerts anti-tumor activity in oncogene-addicted human medullary thyroid cancer
Source: Oncotarget. 2016 Jun 16;7(31):49649–63. doi: 10.18632/oncotarget.10105 (PMC5226536; doi:10.18632/oncotarget.10105)
Supplement: Supplementary file 1 [file oncotarget-07-49649-s001.pdf]

Targeting of *RET* oncogene by naphthalene diimide-mediated gene promoter G-quadruplex stabilization exerts anti-tumor activity in oncogene-addicted human medullary thyroid cancer

SUPPLEMENTARY MATERIALS AND METHODS

Transfection experiments

Transient transfection of RET encoding vectors was carried out according to standard procedures. Briefly, wild-type and MEN2A (C634R)-associated RET pRc/CMVconstructs were obtained as previously described [38]. Twenty-four (MCF-7) or 72 (TT) h after seeding in a 6-well plate, cells ($0.3x10^6$ cells/well) were transiently transfected with 2.5 µg/well of pRC/CMV plasmid (Thermo Fisher Scientific, Monza, Italy) encoding wild-type or mutant (C634R) protoRET using Lipofectamine® 3000 (Thermo Fischer Scientific) in standard growth medium containing 10% FBS, according to the manufacturer's protocol. Forty-eight hours after transfection, cells were treated with the NDI derivative for 48 and 96 h at a concentration corresponding to the IC_{50}. Cells were then collected, counted in a particle counter and subsequently analysed.

SUPPLEMENTARY FIGURES AND TABLES

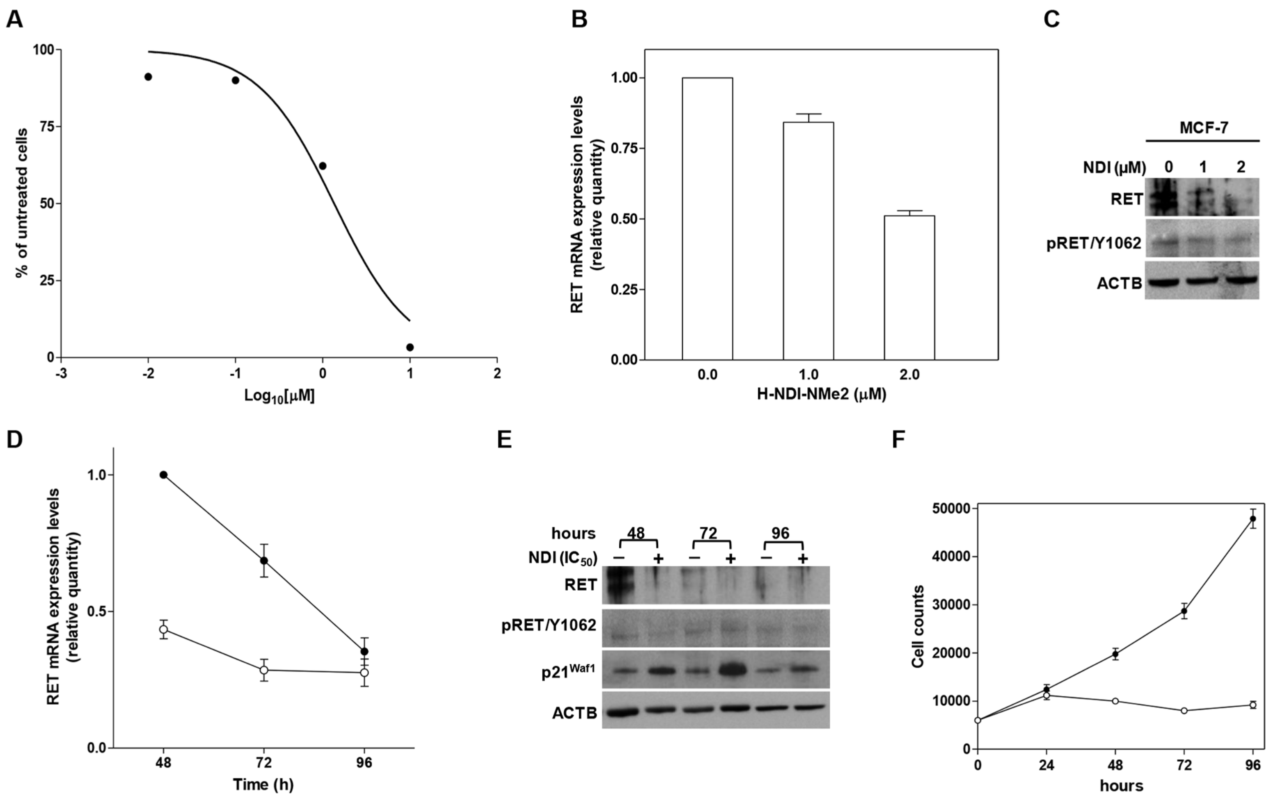

Supplementary Figure S1: Exposure of MCF-7 breast cancer cells to NDI resulted in the impairment of cell growth and in the down-regulation of RET expression. A. Cell growth inhibition curve obtained upon a 48-h exposure to increasing concentrations (Log_{10} [μM]) of NDI. Data have been reported as percentage of growing cells in treated *vs.* untreated cells and represent mean values; **B.** Real-time RT-PCR assessment of RET mRNA expression in MCF-7 cells exposed for 24 h to increasing concentrations of NDI. Quantification of RET mRNA levels was carried out according to the $2^{-\Delta\Delta Ct}$ method [39]. Data have been reported as RET mRNA relative quantity in treated *vs.* untreated cells and represent mean values ± s.d.; **C.** Representative western immunoblotting showing basal and phosphorylated (pRET/Y1062) RET protein amounts in cells exposed for 24 h to the indicated concentrations of NDI. β-actin (ACTB) was used to ensure equal protein loading; **D.** Time-course assessment of RET mRNA expression levels in untreated (●) and NDI-treated (○) cells. Data have been reported as RET mRNA relative amounts in untreated and NDI-treated cells, according to $2^{-\Delta CT}$ method [39], and represent mean values ± s.d.; **E.** Representative western immunoblotting showing the time-course assessment of basal and phosphorylated (pRET/Y1062) RET as well as of $p21^{waf1}$ protein amounts in untreated and NDI-treated MCF-7 cells; **F.** Time-course evaluation of cell growth in untreated (●) and NDI-treated (○) breast cancer cells. Data have been reported as number (cell counts) of growing cells and represent mean values ± s.d..

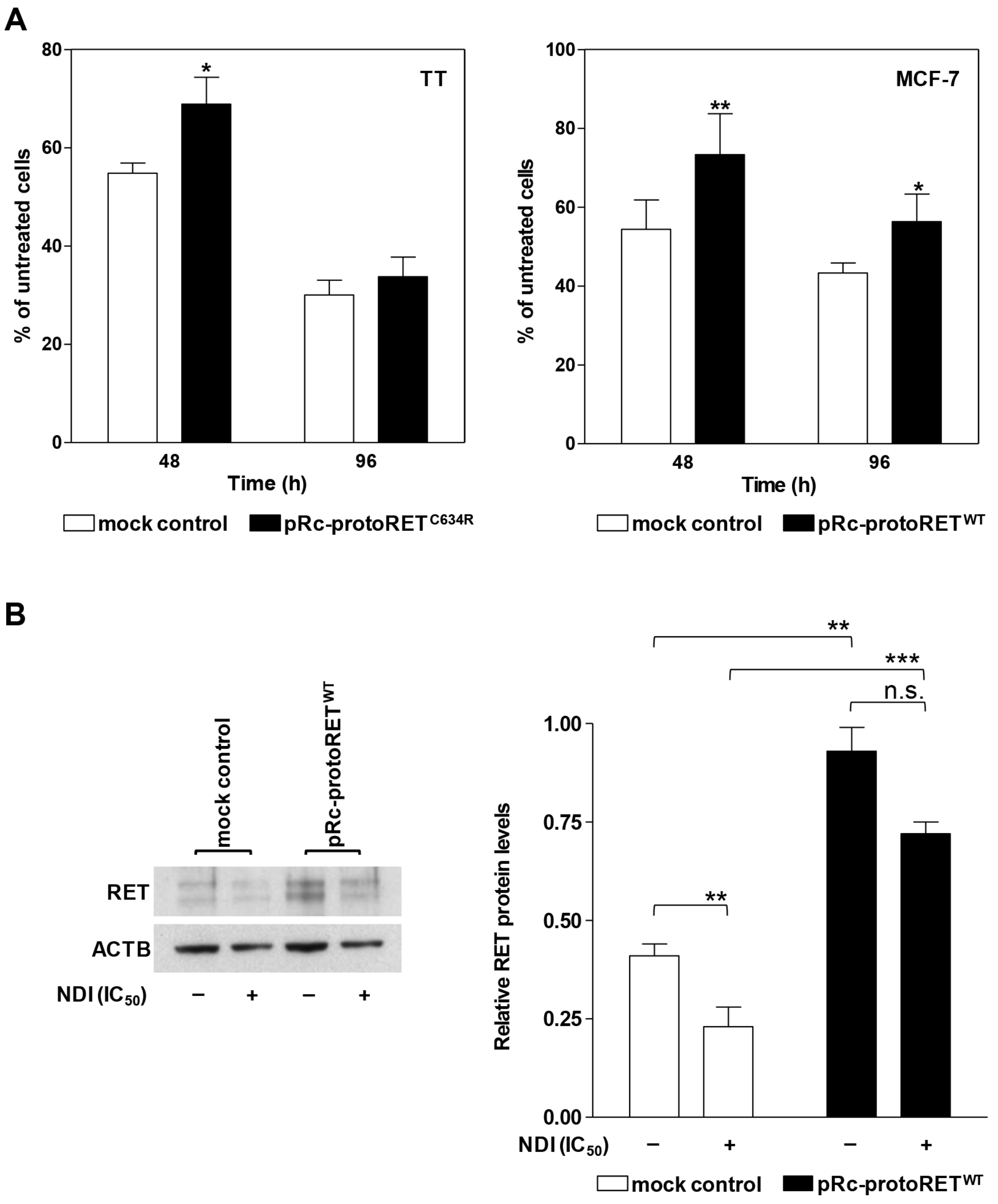

Supplementary Figure S2: The ectopic expression of RET partially counteracts the growth inhibitory effects of NDI in MTC and BCa cells. A. TT (left panel) and MCF-7 (right panel) cells transfected with empty pRc/CMV vector (mock control) and MEN2A-associated (pRc-protoRET^{C634R}) or wild-type (pRc-protoRET^{WT}) RET-encoding vectors were exposed for 48 and 96 h to NDI (IC_{50}). Data have been reported as percentage of growing cells in NDI-treated *vs.* untreated cells and represent mean values ± s.d. *$P<0.05$; **$P<0.01$; **B.** Representative western immunoblotting showing the expression levels of RET in mock control and pRc-protoRET^{WT}–transfected MCF-7 cells in the absence or presence of NDI (IC_{50}). β-actin (ACTB) was used to ensure equal protein loading. The quantification of relative RET protein levels in control and pRc-protoRET^{WT}–transfected MCF-7 cells has been reported in the graph on the right. Data represent mean values ± s.d. **$P<0.01$; ***$P<0.001$.

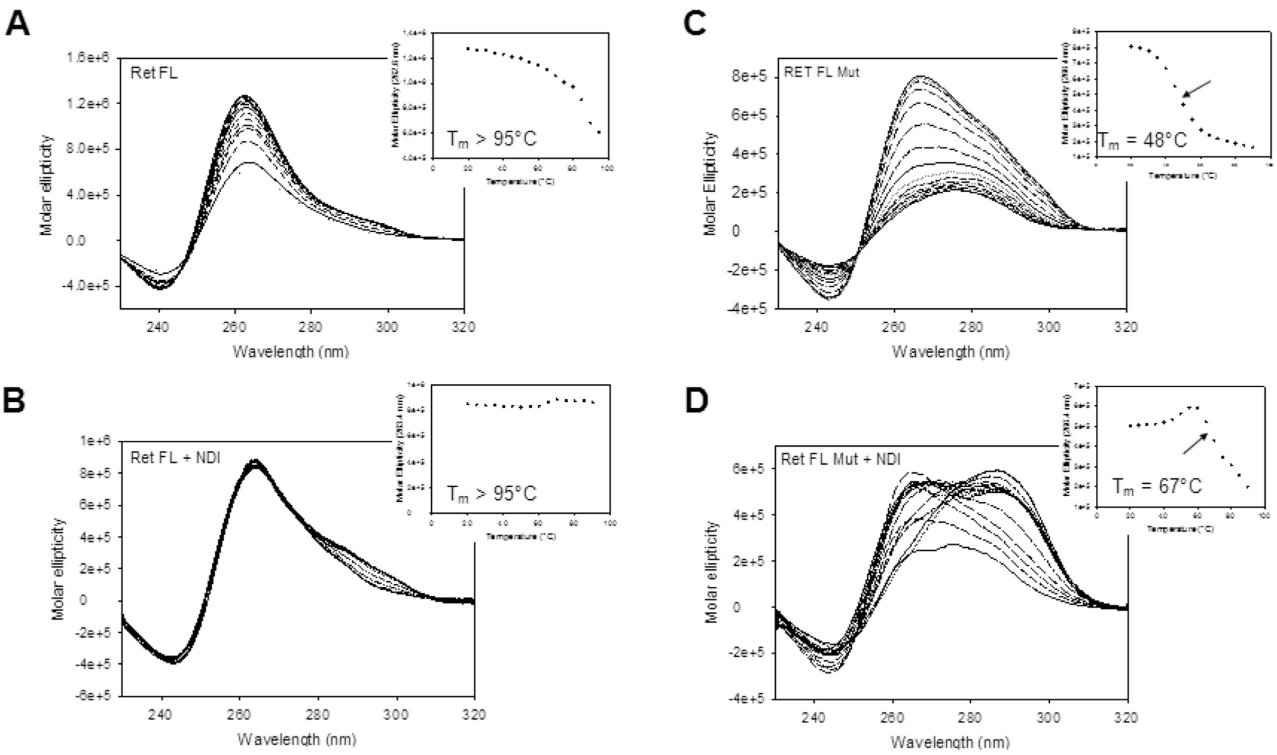

Supplementary Figure S3: CD thermal unfolding analysis of *RET* promoter G4 sequences. A. and **B.** CD spectra of the RET FL oligonucleotide at increasing temperature (25-95 °C) in the absence A) or presence B) of NDI. The insets show molar ellipticity at the indicated wavelength as a function of temperature. **C.** and **D.** CD spectra of the RET FL Mut oligonucleotide at increasing temperature (25-95 °C) in the absence C) or presence D) of the NDI. The insets show molar ellipticity at the indicated wavelength as a function of temperature. T_m values are indicated in the insets.

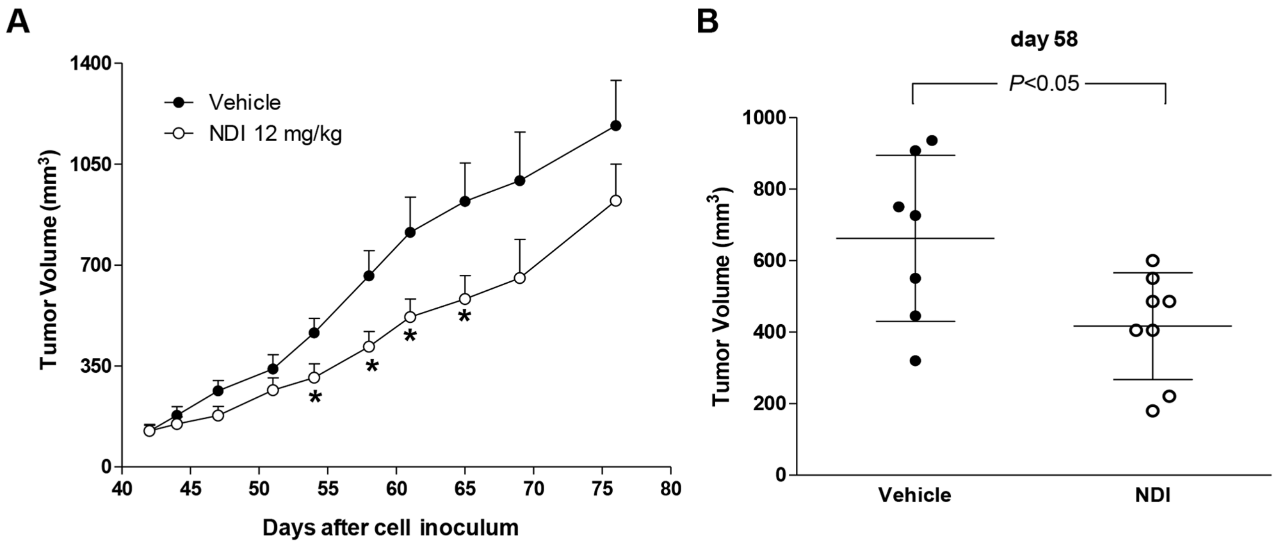

Supplementary Figure S4: The NDI derivative inhibits MTC tumor growth *in vivo*. A. Growth curves of TT tumors in vehicle-treated mice (●) and upon i.p. administration of 12 mg/kg NDI derivative (○). Data have been reported as average tumor volume (mm^3) ± S.E.M. *P<0.05. **B.** Tumor volume distribution in vehicle- and NDI-treated animals at day 58, at which the maximum TVI% was observed.

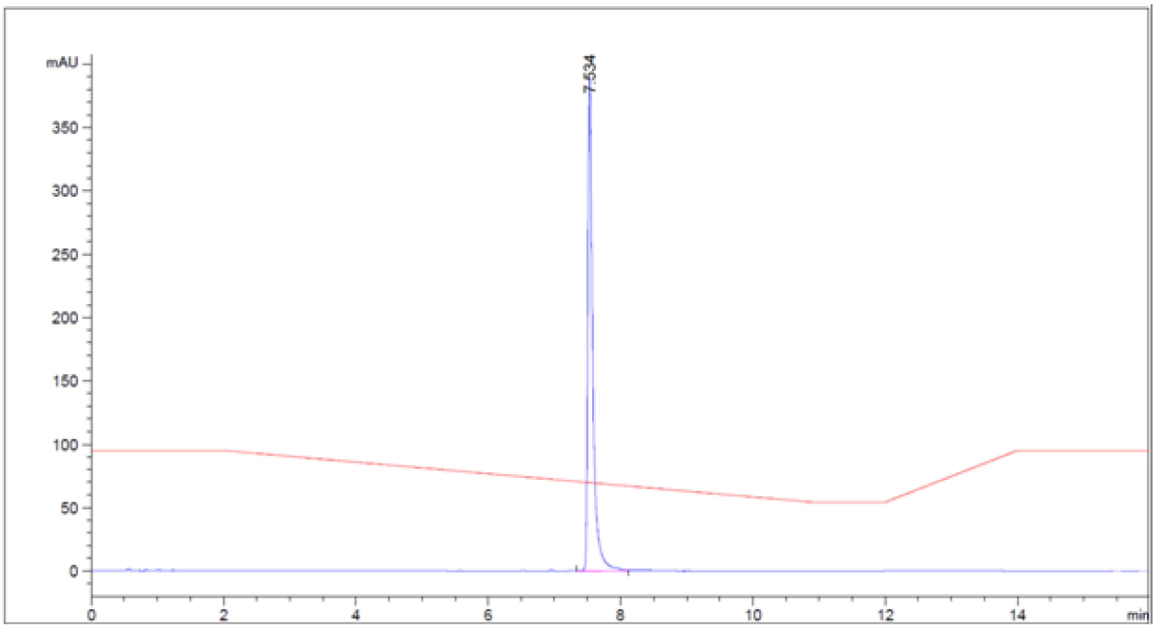

Signal 1: VWD1 A, Wavelength= 256 nm

Peak #	RetTime [min]	Type	Width [min]	Area [mAU*s]	Height [mAU]	Area %
1	7.534	VV	0.0755	2013.92944	388.64218	100.0000
Totals :				2013.92944	388.64218	

Time	% H_2O TFA 0.1%	% CH_3CN
	95	5
**2**	95	5
**11**	54.5	45.5
**12**	54.5	54.5
**14**	95	5
**16**	95	5

Supplementary Figure S5: HPLC purity data of the NDI derivative. The analytical chromatogram of a NDI sample assessed by Method-1 has been reported. HPLC flux 1.0 ml/min.

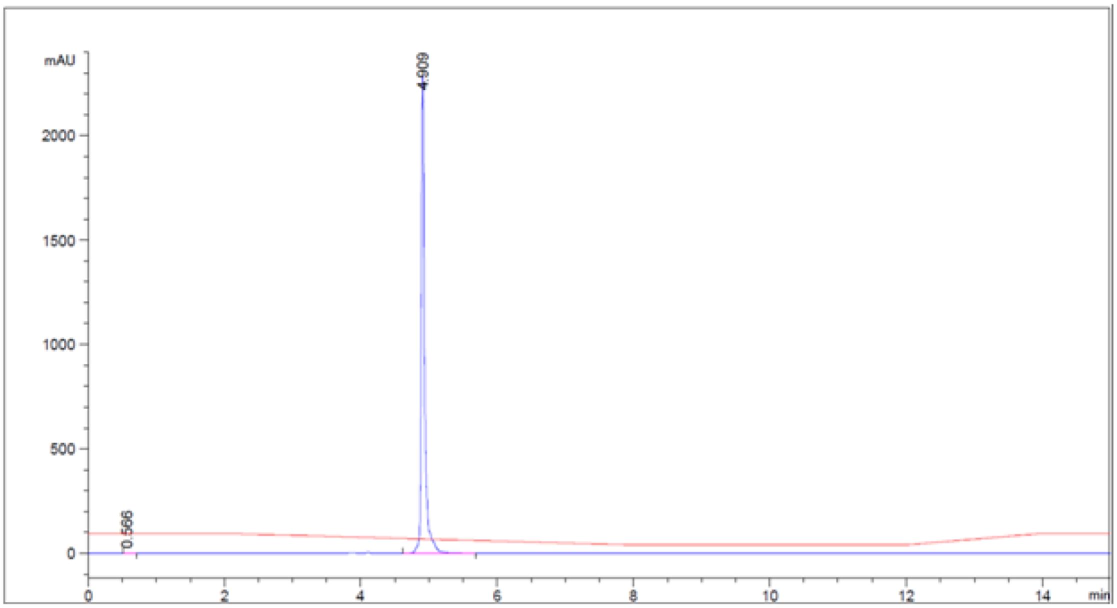

Signal 1: VWD1 A, Wavelength= 256 nm

Peak #	RetTime [min]	Type	Width [min]	Area [mAU*s]	Height [mAU]	Area %
1	0.566	BB	0.0387	5.39401	2.01595	0.0677
2	4.909	VV	0.0521	7959.05518	2287.87793	99.9323
Totals :				7959.05518	2287.87793	

Time	% H_2O TFA 0.1%	% CH_3CN
	95	5
**2**	95	5
**8**	40	60
**12**	40	60
**14**	95	5
**16**	95	5

Supplementary Figure S6: HPLC purity data of the NDI derivative. The analytical chromatogram of a NDI sample assessed by Method-2 has been reported. HPLC flux 1.4 ml/min.

Supplementary Table S1: Oligonucleotides used in this study

Application	Name	Sequence (5′→3′)
CD	RET FL	TTTTTGC**GGG**TA**GGGG**C**GGGG**C**GGGG**C**GGGGG**CGGTTTTT
	RET I-IV	TTTTTA**GGGG**C**GGGG**C**GGGG**C**GGGGG**CGGTTTTT
	RET II-V	TTTTTGC**GGG**TA**GGGG**C**GGGG**C**GGGG**CTTTTT
	RET FL Mut	TTTTTGC**GGG**TA**GGTG**C**GGGG**C**GGAG**C**GGGGG**CGGTTTTT
	RET I-IV Mut	TTTTTA**GGTG**C**GGGG**C**GGAG**C**GGGGG**CGGTTTTT
	RET II-V Mut	TTTTTGC**GGG**TA**GGTG**C**GGGG**C**GGAG**CTTTTT
	c-Myc	T**GGGG**A**GGG**T**GGGG**A**GGG**T**GGGG**AAGG
	c-Kit1	A**GGG**A**GGG**CGCT**GGG**AGGA**GGG**
Taq polymerase stop assay	RET Taq primer	GGCAAAAAGCAGCTGCTTATATGCAG
	RET FL Taq	TTTTT**GCGGG**TA**GGGG**C**GGGG**C**GGGG**C**GGGGG**<br>CGGTTTTTCTGCATATAAGCAGCTGCTTTTTGCC
	RET I-IV Taq	TTTTTA**GGGG**C**GGGG**C**GGGG**C**GGGGG**CGG<br>TTTTTCTGCATATAAGCAGCTGCTTTTTGCC
	RET Mut FL Taq	TTTTTGC**GGG**TA**GGTG**C**GGGG**C**GGAG**C**GGGGG**CGG<br>TTTTTCTGCATATAAGCAGCTGCTTTTTGCC
	RET Mut I-IV Taq	TTTTTA**GGTG**C**GGGG**C**GGAG**C**GGGGG**CGG<br>TTTTTCTGCATATAAGCAGCTGCTTTTTGCC
	Control Taq	TTGTCGTTAAAGTCTGACTGCGAGCTCTCA<br>GATCCTGCATATAAGCAGCTGCTTTTTGCC
Cloning of pGL3-RETmut	Forward primer	GTCAGGTACCGCCCCTTCCCGCACCCCACCCGCCTCCGGCCCCGC<br>CTGGCCCACCCCTGGACCGCCCCCGCTCCGCCCCGCACCTACCCG<br>CTCC
	Reverse primer	TGACCAAGCTTGTTCCGGGGCACTCAGCGCT

G-tracts are shown in bold. Mutated bases are underlined.

Supplementary Table S2: Stabilization of the G4s of *RET*, *KIT1* and *MYC* promoters (4 µM) by the NDI (4 µM) in 10 mM KCl

Sequence	ΔTm (°C)
**RET FL**	1.3 ± 0.5
**RET I-IV**	1.2 ± 0.1
**RET II-V**	5.6 ± 0.4
**c-Kit1**	1.5 ± 0.1
**c-Myc**	3.6 ± 0.3
